# X chromosome dosage and presence of *SRY* shape sex-specific differences in DNA methylation at an autosomal region in human cells

**DOI:** 10.1186/s13293-018-0169-7

**Published:** 2018-02-20

**Authors:** Bianca Ho, Keelin Greenlaw, Abeer Al Tuwaijri, Sanny Moussette, Francisco Martínez, Elisa Giorgio, Alfredo Brusco, Giovanni Battista Ferrero, Natália D. Linhares, Eugênia R. Valadares, Marta Svartman, Vera M. Kalscheuer, Germán Rodríguez Criado, Catherine Laprise, Celia M. T. Greenwood, Anna K. Naumova

**Affiliations:** 1 Department of Human Genetics, McGill University, Montreal, Quebec Canada; 2 Lady Davis Research Institute, Montréal, Quebec Canada; 3 The Research Institute of the McGill University Health Centre (MUHC), 1001 Decarie Blvd., Bloc E, Room EM03226, Montreal, Quebec H4A 3J1 Canada; 4 Unidad de Genética, Hospital Universitario y Politécnico La Fe, 46026 Valencia, Spain; 5 Department of Medical Sciences, University of Torino, 10126 Turin, Italy; 6 Medical Genetics Unit, Città della Salute e della Scienza University Hospital, 10126 Turin, Italy; 7 Department of Public Health and Pediatrics, University of Torino, 10126 Turin, Italy; 8 Setor de Citogenética, Laboratório Central do Hospital das Clínicas da Universidade Federal de Minas Gerais, Belo Horizonte, Brazil; 9 Departamento de Propedêutica Complementar, Faculdade de Medicina, Universidade Federal de Minas Gerais, Belo Horizonte, Brazil; 10 Ambulatório de Erros Inatos do Metabolismo, Hospital das Clínicas da Universidade Federal de Minas Gerais, Belo Horizonte, Brazil; 11 Departamento de Biologia Geral, Instituto de Ciências Biológicas, Universidade Federal de Minas Gerais, Belo Horizonte, Brazil; 12 Research Group Development and Disease, Max Planck Institute for Molecular Genetics, Berlin, Germany; 13 Unidad de Genética Clínica, Hospital Virgen del Rocío, 41013 Sevilla, Spain; 14 Département des Sciences Fondamentales, Université du Québec à Chicoutimi, Chicoutimi, Centre intégré universitaire de santé et services sociaux du Saguenay, Lac-Saint-Jean, Saguenay, Quebec Canada; 15 Centre de santé et de services sociaux de Chicoutimi, Saguenay, Québec Canada; 16 Departments of Oncology and Epidemiology, Biostatistics and Occupational Health, McGill University, Montreal, Quebec Canada; 17 Department of Obstetrics and Gynecology, McGill University, Montreal, QC Canada

**Keywords:** DNA methylation, Sex, X chromosome, Y chromosome

## Abstract

**Background:**

Sexual dimorphism in DNA methylation levels is a recurrent epigenetic feature in different human cell types and has been implicated in predisposition to disease, such as psychiatric and autoimmune disorders. To elucidate the genetic origins of sex-specific DNA methylation, we examined DNA methylation levels in fibroblast cell lines and blood cells from individuals with different combinations of sex chromosome complements and sex phenotypes focusing on a single autosomal region––the differentially methylated region (DMR) in the promoter of the *zona pellucida* binding protein 2 (*ZPBP2*) as a reporter.

**Results:**

Our data show that the presence of the sex determining region Y (*SRY*) was associated with lower methylation levels, whereas higher X chromosome dosage in the absence of *SRY* led to an increase in DNA methylation levels at the *ZPBP2* DMR. We mapped the X-linked modifier of DNA methylation to the long arm of chromosome X (Xq13-q21) and tested the impact of mutations in the *ATRX* and *RLIM* genes, located in this region, on methylation levels. Neither *ATRX* nor *RLIM* mutations influenced *ZPBP2* methylation in female carriers.

**Conclusions:**

We conclude that sex-specific methylation differences at the autosomal locus result from interaction between a Y-linked factor SRY and at least one X-linked factor that acts in a dose-dependent manner.

**Electronic supplementary material:**

The online version of this article (10.1186/s13293-018-0169-7) contains supplementary material, which is available to authorized users.

## Background

Recent genome-wide studies of DNA methylation in human cells have detected hundreds to thousands of autosomal loci where methylation levels differ between the sexes [1–5]. It has been proposed that sex-specific differences in methylation levels contribute to sexual dimorphism in complex phenotypes such as early cognitive processes [4], pancreatic islet function [1], predisposition to asthma [6], and psychiatric disorders [5]. While differences in methylation of X-linked genes are explained by X-chromosome inactivation, the cause of sex-specific methylation of autosomal loci remains elusive with the two main suspects: the sex chromosome complement and gonadal hormones. The goal of this study was to determine if the sex chromosomes contribute to sexual dimorphism in autosomal DNA methylation in human cells.

Mammalian males and females have different sex chromosome complements (XX in females and XY in males). The sex chromosome complement determines gonadal sex, which in turn defines production of gonadal hormones. The ensemble of current evidence suggests that both major factors, the sex chromosome complement and gonadal hormones either acting separately or in concert lead to sexual dimorphism in a wide array of phenotypes (reviewed in [7, 8]).

Sex-specific differences in phenotypes and gene expression patterns have been documented in different species from *Drosophila* to humans ([9–12]. Sexually dimorphic expression is seen in genes residing on the sex chromosomes, as well as autosomal genes. Expression of certain autosomal genes depends on the sex chromosome dosage, whereas other autosomal genes are controlled by sex hormones, as demonstrated in mice and humans [13–16]. In mice, about 10% of autosomal genes have different expression levels between males and females in somatic cells [17]. Most of these differences depend on the sex of the mouse; however, expression levels of hundreds of genes depend on the sex chromosome complement [13]. Sexual dimorphism in autosomal gene expression may result from different doses of transcription factors that are encoded by the sex chromosome-linked genes, especially those X-linked genes that escape X-inactivation. It may also arise as a consequence of sex-specific differences in methylation levels of regulatory elements. In humans, most sexually dimorphic autosomal differentially methylated regions have higher methylation levels in females [1, 2, 18], whereas repetitive elements have higher methylation in males [2, 19–23]. In females with Turner syndrome and monosomy X, global DNA methylation levels are lower than in 46,XX females, but higher than in 46,XY males [24, 25]. This suggests that both sex chromosomes as well as gonadal hormones contribute to the establishment or maintenance of global DNA methylation patterns.

Variation in DNA methylation often correlates with variation in chromatin modifications. Enhancer-specific active chromatin marks, H3K27ac and H3K4me1, co-localize with loci that show sex-specific differences in methylation in the mouse liver [26]. Moreover, the analysis of combined data from the ENCODE and Roadmap Epigenomics projects suggests that sex-specific differences in epigenetic marks are more likely to occur at enhancer regions and “bivalent” regions that carry both active and repressive chromatin marks [27].

To elucidate the genetic mechanisms contributing to sexual dimorphism in DNA methylation, we asked whether the sex chromosome complement influenced DNA methylation levels in human cells (Additional file 1: Figure S1). To address this question, we focused on a single autosomal differentially methylated region (DMR) located within the promoter/enhancer region of the human *zona pellucida* binding protein 2 gene (*ZPBP2*) and used it as a marker DMR. DNA methylation of the *ZPBP2* DMR shows robust differences between males and females in different tissues/cell types [2, 4, 6, 28]. Here, we show that both the Y and X chromosomes control *ZPBP2* methylation levels.

## Methods

### Subjects

DNA samples from the peripheral blood of five carriers of Xq13-q21 duplications with known boundaries of duplicated regions [29, 30] were used for methylation analysis. Three carriers of ATRX, chromatin remodeler (*ATRX*) mutations c.7366_7367insA [31], eight heterozygous carriers of ring finger protein, LIM domain interacting (*RLIM*) gene mutations p.Arg387Cys, and six non-carrier family members were also included in the study [32].

The DNA samples from males and females from the Saguenay-Lac-Saint-Jean (SLSJ) asthma familial collection were used as additional controls to compare methylation levels between males, females, and female carriers of X chromosomal duplications and mutations in X-linked genes [6, 28]. We therefore used *ZPBP2* methylation data from previously published works as a reference for methylation levels in peripheral blood cells (PBC).

All experiments were conducted in accordance with the Canadian Tri-Council Policy Statement of Ethical Conduct for Research Involving Humans and approved by the REB of the MUHC.

### DNA samples from fibroblast cell lines

The DNA samples extracted from untransformed human fibroblast cell lines were purchased from the Coriell Cell Repository (Camden, New Jersey, USA). Information on karyotype, genomic positions of translocation/deletion breakpoints, sex, age, number of passages, and the presence or absence of sex-determining region Y (*SRY*) were obtained from the Coriell Institute for Medical Research website [33]. If no *SRY* information was available, the samples were genotyped for *SRY* by PCR (primers are listed in Additional file 2: Table S1). Cell lines and their characteristics are listed in Additional files 3 and 4: Tables S2 and S3.

## DNA methylation assays

The DNA samples were treated with sodium bisulfite using the EpiTect Bisulfite Kit (Qiagen) per manufacturer’s protocol with modifications. The incubation step was extended by one cycle of 5 min at 95 °C and 2 h at 60 °C before holding at room temperature overnight until purification. The samples were purified according to the manufacturer’s protocol, dried at 56 °C for 5 min, and eluted in 40 μl (2 × 20 μl) of the elution buffer supplied with the kit.

Pyrosequencing methylation assays for the *ZPBP2* promoter/enhancer region were carried as previously described [6, 28]. The assay interrogates 11 CGs located near the transcriptional start site of *ZPBP2* (Additional file 5: Figure S2). This region is referred to as *ZPBP2* DMR from this point on. Methylation was assayed using the PyroMark Q24 Advanced platform (Qiagen) and PyroMark Q24 Advanced CpG Reagents. The assay was conducted using separate sequencing primers (Additional file 2: Table S1). The results were analyzed by the PyroMark Q24 Advanced 3.0.0 software (Qiagen). Sodium bisulfite sequencing assays to interrogate methylation of 51 CGs of the *ZPBP2* promoter region were conducted as previously described [34] (Additional file 5: Figure S2). The *ZPBP2* DMR targeted by pyrosequencing overlaps with CGs 26–35 of the sodium bisulfite sequencing *ZPBP2* methylation assay. Sanger sequencing of clones was performed by the McGill University and Genome Quebec sequencing platform.

### Statistical analysis

To study the influences of phenotypic sex and sex chromosome complement on the methylation patterns in these cell lines, *ZPBP2* DMR methylation was analyzed in 36 fibroblast cell lines: 13 cell lines from 46,XX females, 7 from 46,XY males, 3 from 46,XX males, 4 from 46,XY females, 7 from 45, X females, one from a 49,XYYYY male, and one from a 48/49,XXXY/XXXXY male (Additional file 3: Table S2). The *ZPBP2* pyrosequencing methylation assay interrogates 11 CGs [6]. Inconsistent quality measures were found at CG1, and therefore, 10 CGs were used for further analyses. The data were missing for at least one of the 10 CGs in seven of the 36 assayed cell lines. Therefore, only 29 fibroblast cell lines were included in the principal component (PC) analysis (Additional file 3: Table S2 and Additional file 6: Table S4).

To improve the signal to noise ratio, the first PC of the 10 CGs was calculated to summarize the methylation data. This PC was plotted against methylation data and multiplied by −1 to ensure that the PC was positively correlated with general methylation trends; the resulting oriented first PC was used as the dependent variable in subsequent linear models. Since the methylation levels at the 10 CGs are correlated, to perform separate analyses would lead to unnecessary multiple tests whereas the principal component captures the primary trend across the region.

Considering the available distribution of X and Y chromosome numbers, observations were grouped to compare samples from cell lines with one X chromosome (coded as “0”) to samples with two or more X chromosomes (coded as “1”). The presence of the *SRY* locus was coded as “1,” and absence as “0” (see Table 1). Relationships between the first PC of methylation and sex phenotype, presence of the Y chromosome, presence of the *SRY* region, number of X chromosomes, donor’s age, and number of passages were first explored descriptively by fitting separate linear regressions. Then, stepwise regression was used to obtain our final model, with model selection criterion based on likelihood ratio tests, with *p* = 0.10 for variable removal and *p* = 0.05 for variable inclusion. Likelihood ratio tests were used instead of Wald tests or score tests since the asymptotic convergence is often better in small sample sizes [35]. The starting model used for stepwise regression included covariates: sex, age, passage number, number of Y chromosomes, as well as main effects, and the interaction for the presence of *SRY* and number of X chromosomes.

**Table 1:** Counts of samples by *SRY* and number of X chromosomes in fibroblast cell lines

Number of X chromosomes	One	Two or more
*SRY* absent	3	12
*SRY* present	11	3

## Results

The presence of the *SRY* region and the number of X chromosomes influence *ZPBP2* methylation levels.

**Fig. 1:**
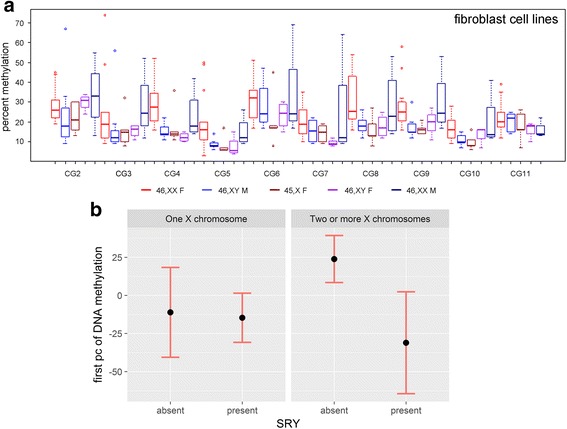
Effect of sex and the sex chromosome complement on *ZPBP2* methylation levels. **a** Methylation levels of 10 CGs located within the *ZPBP2* promoter region in fibroblast cell lines from 46,XX females, 46,XY males, 45,X females, 46,XY females, and 46,XX males. **b** Results of linear regression analysis showing means and 95% confidence intervals of the first principal component, averaged over the two sexes. The differences are interpretable as changes in percent methylation, although the exact values are intercept adjusted. Left: one X chromosome; right: 2 or more X chromosomes. Within each panel, *SRY* is denoted as absent or present

To test the impact of the sex-chromosome complement on autosomal DNA methylation levels in human cells, as a first step, we used human non-transformed fibroblast cell lines derived from donors with different combinations of sex-chromosome complement and sex that would permit separating the effects of sex chromosomes from those of sex phenotype (Additional file 3: Table S2 and Additional file 1: Figure S1). We selected the *ZPBP2* DMR located within the *ZPBP2* promoter as a marker of sex-specific differences in methylation levels. Previous studies showed robust sex-specific differences in DNA methylation in PBC, higher methylation levels in females, and differences in methylation greater than 5% in this region [2, 4, 6, 28]. We analyzed *ZPBP2* DMR methylation levels in 36 fibroblast cell lines with different combinations of sex chromosome complement and sex phenotype (Fig. 1a). Of these, 29 cell lines had complete data for the 10 CGs of the *ZPBP2* DMR and were used for PCA: these included 11 cell lines from 46,XX females; 4––from 46,XY males; 3––from 46,XX males; 3––from 46,XY females, 6––from 45,X individuals; one from a 49,XYYYY male, and one from a 48/49,XXXY/XXXXY male (Additional file 7: Figure S3 and Additional file 6: Table S4).

Methylation levels of the *ZPBP2* DMR in fibroblast cell lines, summarized by their first PC, were analyzed to examine different combinations of sex phenotype and sex chromosome complement (Fig. 1b). The first PC captures 59.2% of the total variation (Additional file 7: Figure S3). After grouping the independent variables of interest, there were 14 samples with one X chromosome and 15 with two or more X chromosomes, and there were 15 samples with *SRY* and 14 samples without *SRY*.

A number of initial linear models were fit to provide a general description of the effects of potential covariates on methylation. When sex phenotype, passage number, number of Y chromosomes, and age were, one at a time, regressed on the first PC of methylation, none of them showed a significant association (*p* = *0.88* for sex, *p* = *0.25* for passage number, *p* = *0.21* for number of Y chromosomes, *p* = *0.65* for age; see also Additional file 8: Table S5). In contrast, a linear model that regressed, individually, the number of X chromosomes and *SRY* on the first PC of methylation showed significant associations (*p* = *0.019* for number of X chromosomes, *p* = *0.037* for *SRY*).

Stepwise regression, using likelihood ratio tests, to choose variables for inclusion and exclusion selected a final model with covariates sex, *SRY*, number of X chromosomes, and an interaction between the latter two variables (Table 2 and Fig. 1b). All multiple regression models excluded one sample with ambiguous sex, and therefore, sample size was 28 in these models. The significant interaction between the number of X chromosomes and *SRY* presence on the methylation patterns is such that the relationship between methylation and the number of X chromosomes is mainly evident when *SRY* is absent (Fig. 1b). In the absence of *SRY*, methylation was higher in the samples with two or more X chromosomes, when compared to a reference group of samples with one X chromosome (Fig. 1b). In the presence of *SRY*, however, the number of X chromosomes appears to have little influence on *ZPBP2* methylation (Table 3), although only three samples with *SRY* and two or more X chromosomes were available for analysis. We realize that due to the numbers of non-transformed fibroblast samples available for this analysis that power is limited (see Table 1 and Table S5); nevertheless, given that only one multiple linear model was fit, no adjustments for multiple comparisons were performed.

**Table 2:** Multiple linear regression results between the first PC of methylation data and covariates

Covariate	Estimate	Std. error	*t* value	Pr(>|t|)
(Intercept)	− 19.44	13.63	− 1.426	0.167
Sex: male	25.90	12.78	2.027	0.054
Presence of *SRY*	− 3.542	16.81	− 0.211	0.835
Two or more X chromosomes	35.00	15.28	2.291	0.032
Interaction: *SRY*: two or more X chromosomes	− 51.38	22.41	− 2.293	0.031

**Table 3:** Differences from average methylation as a function of number of X chromosomes and *SRY*

*SRY*	Number of X chromosomes	
	One	Two or more
Absent	− 11.11	23.89
Present	− 14.65	− 31.03

Stepwise multiple linear regression results, chosen by comparing model fits with likelihood ratio tests, between the first PC of methylation data at *ZPBP2* and covariates. Estimates and standard errors are in units of methylation percentages. The number of samples in all analyses is 28. This model has an adjusted R-squared of 0.30 and a naïve R-squared of 0.41. Note that the *p* value of 0.054 for “sex” is obtained from a Wald test; this variable was retained in the model based on a likelihood ratio test with *p* = 0.032.

The results are estimated from the multiple linear regression in Table 2 and averaged over the two sexes.

### The X-linked modifier of DNA methylation maps to chromosomal region Xq13-Xq21

Since the X chromosome dosage influenced methylation, the next step was to map the region of the X chromosome harboring the gene(s) responsible for this influence. We reasoned that the X-linked modifier of DNA methylation should satisfy the following criteria: (i) two or more copies of the modifier would result in higher methylation levels compared to one copy and (2) loss of function mutations in the modifier in 46,XX females would result in lower methylation levels at the reporter locus.

**Fig. 2:**
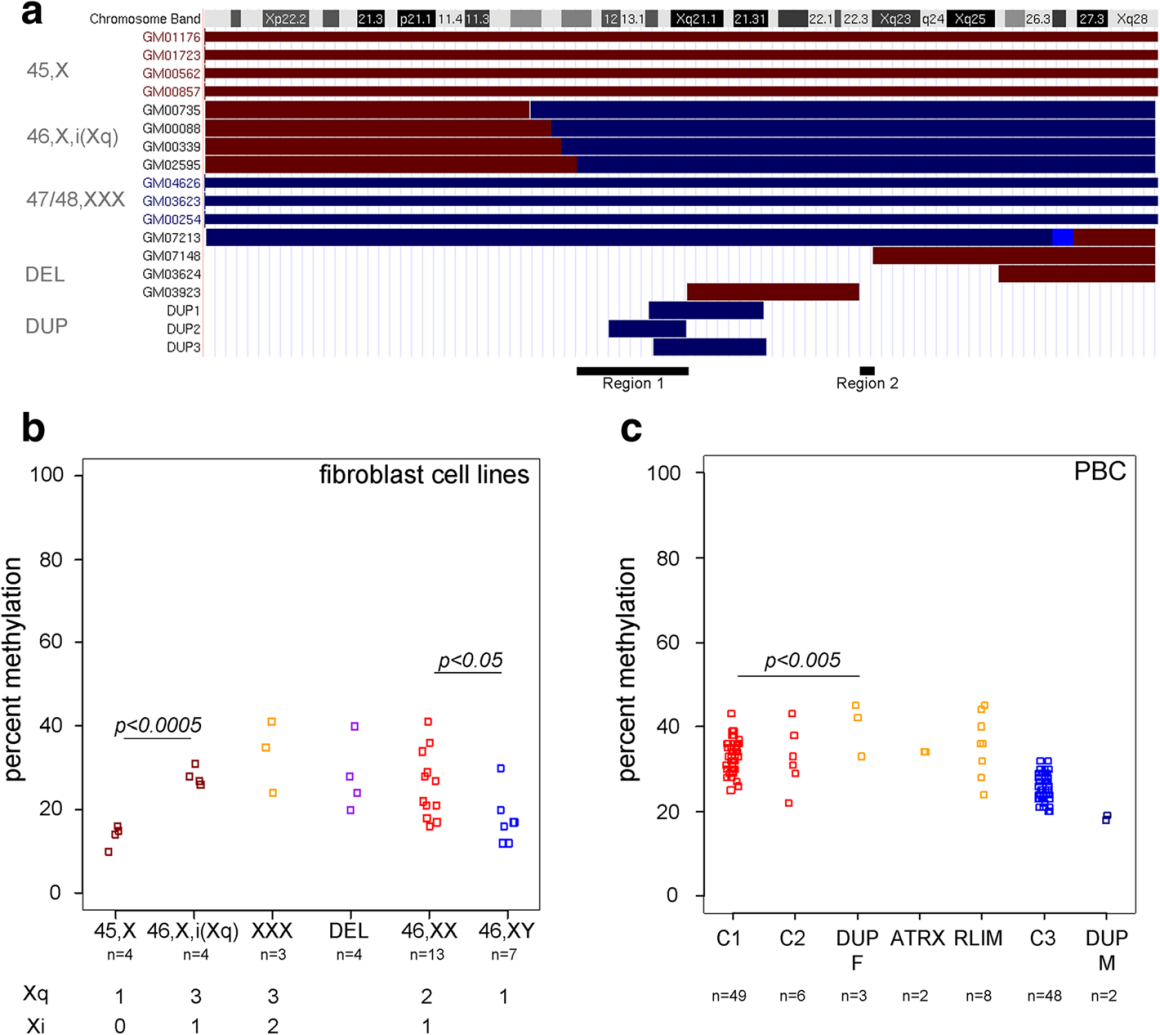
The X-linked modifier of *ZPBP2* methylation resides on the q-arm of the X chromosome. **a** Dosage of X-chromosomal regions in female fibroblast cell lines and female carriers of Xq10-q21 duplications tested in the study. Regions present in one copy are shown in dark red. Regions present in three copies are shown in dark blue and in four to five copies in blue. The rest are present in two copies. The cell line ID is shown on the left. The coordinates of the deletion and duplication boundaries are based on array-CGH data. The possible locations of the X-linked gene that influences *ZPBP2* methylation are shown below the diagram. **b** Average methylation levels across the 10 CGs of the *ZPBP2* DMR in fibroblast cell lines from Turner syndrome patients with an 45,X karyotype (*n* = 4) compared to Turner syndrome patients with a 46,X,i(Xq) karyotype (*n* = 4), carriers of Xq deletions (*n* = 4), female carriers of trisomy X (47/48,XXX) (*n* = 3), 46,XX females (*n* = 13), and 46, XY males (*n* = 7). Numbers of samples, copies of the Xq, and numbers of Xi are shown below the *x*-axis. **c** Average methylation levels across the 10 CGs of the *ZPBP2* DMR in peripheral blood cells (PBC) from females (C1, *n* = 49), non-carrier female relative of *RLIM* mutation carriers (C2, *n* = 6), heterozygous female carriers of Xq13-q21 duplications (*n* = 3), heterozygous female carriers of an *ATRX* mutation (*n* = 2), heterozygous female carriers of an *RLIM* mutation (*n* = 8), males (C3, *n* = 48), and male carriers of Xq13-q21 duplications (*n* = 2)

We first asked if the X-linked modifier resided on the Xp or Xq arms and compared *ZPBP2* methylation in fibroblast cell lines from four Turner syndrome patients with monosomy X (45, X), four individuals with monosomy for most of the p-arm and trisomy for the q-arm of X chromosome (referred to as 46,X,i(Xq)) and three females with trisomy X (47 or 48,XXX) (Fig. 2a). Since the *SRY* region may reduce methylation levels in samples with two or more X chromosomes, we selected cell lines in which *SRY* was absent. The methylation levels at individual CGs within the *ZPBP2* DMR were correlated (Pearson correlation coefficient ranging from 0.36 to 0.85). Hence, we used the average methylation levels across the 10 CGs as a read-out. Fibroblast cell lines from Turner syndrome patients with monosomy X had significantly lower methylation levels than those with three copies of the Xq (*p = 0.00013*, one-tailed Student’s *t* test) (Fig. 2b). A similar trend was observed in the extended *ZPBP2* promoter region (Additional file 5: Figure S2B). Therefore, at least one of the genes that influence *ZPBP2* DMR methylation resides on the long arm of the X chromosome.

To test the possibility that the number of inactive X chromosomes rather than the dose of a particular gene influenced *ZPBP2* DMR methylation levels, we compared females with two inactive X chromosomes (Xi) and three copies of Xq (47/48,XXX) to those with one Xi and three copies of Xq (46,X,i(Xq)). No significant differences in methylation levels were found between these two groups (*p* = *0.13*, one-tailed Student’s *t* test). Methylation in 46,X,i(Xq) samples was not statistically different from that in 46,XX females (*p* = *0.49*, two-tailed Student’s *t* test).

Next, to further reduce the candidate region for the Xq-linked methylation modifier(s), we examined four fibroblast cell lines from females with different Xq deletions (Fig. 2a). *ZPBP2* methylation levels in these cell lines were higher than in 45,X Turner syndrome patients (Fig. 2b). We hypothesized that all four cell lines with Xq deletions contained two or more copies of the X-linked modifier. Two Xq regions, referred to as region 1 and region 2 from this point on, were present in all four cell lines in more than one copy (Fig. 2a). Therefore, if there was only one methylation modifier in Xq, it had to reside in one of these two regions. Region 1 spans more than 10 Mb between the centromere and genomic position 78.6 Mb (hg19) (Xq11-q21) and includes the X-inactivation center. Region 2 spans a 2.2 Mb genomic interval between positions 106.6 and 108.8 Mb (Xq22.3) of the X chromosome and includes 18 annotated genes.

We next hypothesized that if region 1 harbored the X-linked modifier of methylation, female carriers of chromosomal duplications of this region would have higher methylation levels than females without duplications. There were no fibroblast cell lines with duplications of the Xq11-q21 region available to us. We therefore assayed *ZPBP2* methylation in peripheral blood of three females who carried duplications of chromosomal region Xq13.2-q21.1 [29, 30] and compared their methylation levels to those observed in the blood of control females and males from the SLSJ familial collection [6] (Fig. 2c). Methylation in the duplication carriers was at the higher end of the range observed in non-carrier females, and the difference reached statistical significance in our sample (*p* = *0.0045*, two-tailed Student’s *t* test) (Fig. 2c). Based on the boundaries of these duplications, we speculate that the candidate gene may reside within a 5.2 Mb genomic interval between genomic positions 73.1 and 78.4 (hg19) **(**Fig. 2a); however, this conclusion must be validated using a larger sample size.

Two genes, the ATRX, chromatin remodeler (*ATRX*) and ring finger protein, LIM domain interacting (*RLIM*) reside in region 1 and are of particular interest because they encode transcription factors that are involved in epigenetic silencing and X chromosome inactivation [36–40]. These genes are subject to X-inactivation [41], but could act before its onset in early embryos. In such a case, the mutation would have an effect independent of whether it resided on the X chromosome that was preferentially inactivated. We therefore hypothesized that if *ATRX* or *RLIM* influenced *ZPBP2* methylation, their mutations would reduce methylation levels in heterozygous carrier females. *ZPBP2* methylation levels were assayed in two female carriers of the disease-causing *ATRX* mutation c.7366_7367insA [31] and eight heterozygous carrier females of the disease-causing mutation p.Arg387Cys in the zinc-finger domain of *RLIM* [32]. The samples from six female relatives of the *RLIM* mutation carriers were also assayed. *ZPBP2* methylation levels in female carriers of *RLIM* and *ATRX* mutations were not different from those found in controls (Fig. 2c). Thus, neither *ATRX* nor *RLIM* mutations reduced the methylation levels of *ZPBP2* in heterozygous females in our study.

## Discussion

Here, we report that X-chromosome dosage and sex determining factor *SRY* region contribute to sexual dimorphism in DNA methylation at an autosomal region, the promoter/enhancer of the *ZPBP2* gene located in chromosomal region 17q12, whereas phenotypic sex and age have no significant effect on methylation. The presence of the whole Y-chromosome or its part harboring the *SRY* gene were associated with lower methylation levels, independent of the number of X chromosomes. To the best of our knowledge, this is the first study of the effect of sex chromosomes and sex phenotype on autosomal methylation in human cells.

In the DNA samples from Turner syndrome patients that carried only one X, the *ZPBP2* DMR methylation levels were lower than in individuals who carried two or more copies of intact X chromosomes, or the Xq arms. Our findings are consistent with hypomethylation of the *ZPBP2* promoter region in the blood cells of Turner syndrome patients compared to 46,XX females [24, 25]. The caveat here is that our conclusions are based on the assumption that rearrangements and aneuploidy for chromosomes other than the sex chromosomes are unlikely to affect *ZPBP2* methylation levels. This assumption has not been tested.

To identify the X-linked genes responsible for the levels of DNA methylation at the *ZPBP2* locus, we compared methylation in fibroblast cell lines from Turner syndrome patients with karyotypes 45,X and 46,X,i(Xq), females with trisomy X, as well as females with deletions within the Xq arm. To confirm the effect of the Xq13-q21 dosage on *ZPBP2* methylation, we used the DNA samples from peripheral blood cells of female carriers of Xq13-q21 duplications. The striking differences in methylation between Turner syndrome patients with one and three copies of Xq suggest that at least one X-linked modifier of *ZPBP2* methylation levels resides on the q-arm of the X chromosome. *ZPBP2* methylation in the samples from Xq13-q21 duplication carriers was at the higher end of the range of controls, but the difference was not as dramatic as the difference between males and females. Therefore, using samples from female carriers of X chromosomal deletions may prove to be a more informative mapping approach.

The results of testing two candidate genes from region 1, *ATRX* and *RLIM*, did not support the hypothesis that the dosage of either one of these genes was responsible for the higher methylation of the *ZPBP2* DMR in females. However, it is possible that a missense mutation in *ATRX* or *RLIM* may impact DNA methylation differently than a deletion of the entire gene would.

In principle, the X-linked gene(s) that is responsible for the dose-dependent effect on DNA methylation should either escape X-chromosome inactivation or act early in embryonic development before the onset of X-inactivation (reviewed in [42]). In humans, sex differences in DNA methylation are present in newborns [4] whereas the earliest developmental stage with sex differences in human gene expression is represented by embryonic stem cells [43]. Sex differences in gene expression are detected in embryonic stem cells derived from mouse blastocysts and in bovine blastocysts and as early as 8-cell stage in mouse embryos [44–46]. Hence, one cannot rule out the possibility that the causal X-linked gene contributes to the establishment of sexually dimorphic DNA methylation patterns before the blastocyst stage, and silencing of one of its copies later in development does not alter DNA methylation patterns. Alternatively, if the sexual dimorphism in DNA methylation is established after the onset of X-inactivation or the X-linked factor is required for the maintenance of higher methylation levels in females, the causal X-linked gene must escape X-inactivation to act in a dose-dependent fashion. Indeed, the X-linked gene for lysine demethylase 5C (*KDM5C*) that escapes X-inactivation and its Y-homolog *KDM5D*, both influence DNA methylation at certain autosomal regions in a dose-dependent fashion [47]. *KDM5C* resides on the short arm of the X chromosome and in principle may be responsible for the tendency to lower methylation in 46,X,i(Xq) compared to trisomy X females.

None of region 2 genes are known to escape X-inactivation. Region 1 is larger, and several of its genes escape X-inactivation including several long non-coding RNAs (e.g., X-inactive specific transcript (*XIST*), JPX transcript, *XIST* activator (*JPX*)) [41, 48]. Region 1 also harbors several micro RNAs whose inactivation status is not known [41, 48]. This warrants further studies of the role of region 1 genes in the establishment of autosomal methylation patterns.

X-chromosome inactivation requires epigenetic silencing of hundreds of genes on the same X chromosome. It has been suggested that the presence of an inactive X per se may influence the genomic distribution of heterochromatic proteins, acting as an epigenetic “sink” or “tank” [13, 49–51]. The data from the studies of meiotic silencing in the mammalian germ line show that, indeed, sex chromosomes and autosomes may compete for silencing factors in certain cell types [52–55] landing additional support for this hypothesis. If this were the mechanism by which X chromosome dosage affected autosomal methylation, it would cause differences in DNA methylation levels between individuals who carry an inactive X and those who do not. Moreover, one would predict that the number of inactive X chromosomes rather than number of copies of a particular X-linked gene would be critical. That is, the presence of an inactive isochromosome Xq would have the same impact on methylation as would one Xi or an Xi with a duplication have. Although our data are consistent with the possibility that the inactive X promotes higher DNA methylation levels at the *ZPBP2* DMR (acting as a “supply center” rather than a “sink” for heterochromatic factors), higher methylation in duplication carriers who have only one Xi, favors the scenario with genes on the Xp and Xq influencing methylation rather than an effect of an inactive X being present.

In contrast to the X chromosome, the presence of the Y-chromosome reduces the level of DNA methylation at the *ZPBP2* DMR and makes it insensitive to the X chromosome dosage. The *SRY* region recapitulates the effect of the whole Y in our small sample of fibroblast cell lines. SRY is a transcription factor that plays a critical role in sex determination (reviewed in [56]). Moreover, the mouse *Sry* has been shown to contribute to sexual dimorphism in autosomal gene expression levels [13]. It has been also implicated as the cause of sexual dimorphism in human brain development [57] and Hirschsprung disease [58]. The mechanism of the SRY effect on methylation has yet to be discovered; however, it is worth noting that our reporter gene *ZPBP2* is highly expressed in testis and implicated in male fertility [59]. We therefore hypothesize that SRY binds the *ZPBP2* promoter and shields it from DNA methylation, thereby preserving its activity in males, in the germ line, and in somatic cells. Such a mechanism would be beneficial for male fertility and explain the enrichment of spermatogenesis-associated genes among those with sex-specific promoter methylation [2, 4]. Thus, although *SRY* resides within a gene-rich region of the Y chromosome and, in our study, its effect cannot be separated from the effects of closely linked genes, it represents a good candidate whose role needs further investigation.

Interestingly, in *Drosophila*, sexual dimorphism in body size is regulated by the dosage of the X-linked gene *Myc*, which is not subject to dosage compensation, and the sex-determining gene transformer (*tra*) [10]. Mouse studies dissecting the mechanism underlying sexual dimorphism in gene expression levels suggest contribution of X- and Y- encoded factors as well as sex hormones [13]. Thus, the sum of current data suggests that the paradigm implicating interaction between X-chromosome-linked and sex-determining genes in sexual dimorphism in insects [10, 60, 61] and mice [13] may also explain some instances of sexually dimorphic autosomal DNA methylation in humans. We focused our study of sexual dimorphism in DNA methylation at a single autosomal locus. However, our findings may reflect a more general rule and larger genomic studies may find similar regulation of methylation at other autosomal loci.

## Conclusions

Our data demonstrate that interaction between the Y chromosome and the X chromosome dosage defines DNA methylation levels at the autosomal locus *ZPBP2*. Furthermore, our findings suggest that at least one X-linked gene that influences *ZPBP2* DMR methylation levels resides on the long arm of chromosome X. This is the first study that attempts dissecting the genetic mechanisms underlying sex-specific differences in methylation levels in a human autosomal region, and our findings may be applicable to other loci.

## Additional files

Additional file 1: **Figure S1.** Experimental design. (DOCX 18 kb)

Additional file 2: **Table S1.** Primers used in the study. (DOCX 12 kb)

Additional file 3: **Table S2.** Fibroblast cell lines used for exploratory analysis. (DOCX 16 kb)

Additional file 4: **Table S3.** Fibroblast cell lines used for mapping of the X-linked modifier of methylation. (DOCX 14 kb)

Additional file 5: **Figure S2.** Sodium bisulfite sequencing methylation analysis of 51 CGs across the *ZPBP2* promoter region shows higher methylation levels in fibroblast cell lines with two and three copies of the Xq arm. A: location of the 51 CGs interrogated using the sodium bisulfite sequencing assay shown in the context of the UCSC browser (hg19). B: heatmap representing mean methylation levels for each of the 51 CGs in cell lines with one X chromosome (two fibroblast cell lines with karyotype 45,X and no *SRY* region) and two or three copies of Xq (data from four fibroblast cell lines with karyotype 46,XX and two fibroblast cell lines from Turner syndrome patients with karyotype 46,i(Xq)). The black box beneath the heatmap shows the location of the 10 CGs analyzed by the pyrosequencing assay. The color scale for percent methylation is shown on the right. (DOCX 292 kb)

Additional file 6: **Table S4.** Methylation percentages at the analyzed *ZPBP2* DMR CGs from human non-transformed fibroblast cell lines, and the first PC of these values. (DOCX 14 kb)

Additional file 7: **Figure S3.** Scatter plot of the first (PC1) and second (PC2) eigenvectors from a principal component analysis of methylation levels at 10 CpGs near the *ZPBP2* transcriptional start site. The first principal component explains 59.2% of the variance in methylation levels. Points are colored by groups defined by presence of SRY and the number of X chromosomes. (DOCX 118 kb)

Additional file 8: **Table S5.** Univariate analysis results, full model multivariate analysis results, as well as post hoc power calculations. (DOCX 14 kb)

## Abbreviations

ATRX: ATRX, chromatin remodeler
DMR: Differentially methylated region
JPX: JPX transcript, *XIST* activator
PBC: Peripheral blood cells
PC: Principal component
PCA: Principal component analysis
RLIM: Ring finger protein, LIM domain interacting
SLSJ: Saguenay-Lac-Saint-Jean
SRY: Sex determining region Y
XIST: X-inactive specific transcript
ZPBP2: *zona pellucida* binding protein 2

## References

[1] Hall E, Volkov P, Dayeh T, Esguerra JL, Salo S, Eliasson L, Ronn T, Bacos K, Ling C (2014). Sex differences in the genome-wide DNA methylation pattern and impact on gene expression, microRNA levels and insulin secretion in human pancreatic islets. Genome Biol.

[2] McCarthy NS, Melton PE, Cadby G, Yazar S, Franchina M, Moses EK, Mackey DA, Hewitt AW (2014). Meta-analysis of human methylation data for evidence of sex-specific autosomal patterns. BMC Genomics.

[3] Xu H, Wang F, Liu Y, Yu Y, Gelernter J, Zhang H (2014). Sex-biased methylome and transcriptome in human prefrontal cortex. Hum Mol Genet.

[4] Yousefi P, Huen K, Dave V, Barcellos L, Eskenazi B, Holland N (2015). Sex differences in DNA methylation assessed by 450 K BeadChip in newborns. BMC Genomics.

[5] Maschietto M, Bastos LC, Tahira AC, Bastos EP, Euclydes VL, Brentani A, Fink G, de Baumont A, Felipe-Silva A, Francisco RP (2017). Sex differences in DNA methylation of the cord blood are related to sex-bias psychiatric diseases. Sci Rep.

[6] Naumova AK, Al Tuwaijri A, Morin A, Vaillancout VT, Madore AM, Berlivet S, Kohan-Ghadr HR, Moussette S, Laprise C (2013). Sex- and age-dependent DNA methylation at the 17q12-q21 locus associated with childhood asthma. Hum Genet.

[7] Burgoyne PS, Arnold AP (2016). A primer on the use of mouse models for identifying direct sex chromosome effects that cause sex differences in non-gonadal tissues. Biol Sex Differ.

[8] Arnold AP, Cassis LA, Eghbali M, Reue K, Sandberg K (2017). Sex hormones and sex chromosomes cause sex differences in the development of cardiovascular diseases. Arterioscler Thromb Vasc Biol.

[9] Ghosh S, Klein RS (2017). Sex drives dimorphic immune responses to viral infections. J Immunol.

[10] Mathews KW, Cavegn M, Zwicky M (2017). Sexual dimorphism of body size is controlled by dosage of the X-chromosomal gene Myc and by the sex-determining gene tra in drosophila. Genetics.

[11] Ecker S, Chen L, Pancaldi V, Bagger FO, Fernandez JM, Carrillo de Santa Pau E, Juan D, Mann AL, Watt S, Casale FP (2017). Genome-wide analysis of differential transcriptional and epigenetic variability across human immune cell types. Genome Biol.

[12] Warnefors M, Mossinger K, Halbert J, Studer T, VandeBerg JL, Lindgren I, Fallahshahroudi A, Jensen P, Kaessmann H (2017). Sex-biased microRNA expression in mammals and birds reveals underlying regulatory mechanisms and a role in dosage compensation. Genome Res.

[13] Wijchers PJ, Yandim C, Panousopoulou E, Ahmad M, Harker N, Saveliev A, Burgoyne PS, Festenstein R (2010). Sexual dimorphism in mammalian autosomal gene regulation is determined not only by Sry but by sex chromosome complement as well. Dev Cell.

[14] Miller VM, Garovic VD, Kantarci K, Barnes JN, Jayachandran M, Mielke MM, Joyner MJ, Shuster LT, Rocca WA (2013). Sex-specific risk of cardiovascular disease and cognitive decline: pregnancy and menopause. Biol Sex Differ.

[15] Irizar H, Munoz-Culla M, Sepulveda L, Saenz-Cuesta M, Prada A, Castillo-Trivino T, Zamora-Lopez G, Lopez de Munain A, Olascoaga J, Otaegui D (2014). Transcriptomic profile reveals gender-specific molecular mechanisms driving multiple sclerosis progression. PLoS One.

[16] Jansen R, Batista S, Brooks AI, Tischfield JA, Willemsen G, van Grootheest G, Hottenga JJ, Milaneschi Y, Mbarek H, Madar V (2014). Sex differences in the human peripheral blood transcriptome. BMC Genomics.

[17] van Nas A, Guhathakurta D, Wang SS, Yehya N, Horvath S, Zhang B, Ingram-Drake L, Chaudhuri G, Schadt EE, Drake TA (2009). Elucidating the role of gonadal hormones in sexually dimorphic gene coexpression networks. Endocrinology.

[18] Mamrut S, Avidan N, Staun-Ram E, Ginzburg E, Truffault F, Berrih-Aknin S, Miller A (2015). Integrative analysis of methylome and transcriptome in human blood identifies extensive sex- and immune cell-specific differentially methylated regions. Epigenetics.

[19] El-Maarri O, Becker T, Junen J, Manzoor SS, Diaz-Lacava A, Schwaab R, Wienker T, Oldenburg J (2007). Gender specific differences in levels of DNA methylation at selected loci from human total blood: a tendency toward higher methylation levels in males. Hum Genet.

[20] El-Maarri O, Walier M, Behne F, van Uum J, Singer H, Diaz-Lacava A, Nusgen N, Niemann B, Watzka M, Reinsberg J (2011). Methylation at global LINE-1 repeats in human blood are affected by gender but not by age or natural hormone cycles. PLoS One.

[21] Vaissiere T, Hung RJ, Zaridze D, Moukeria A, Cuenin C, Fasolo V, Ferro G, Paliwal A, Hainaut P, Brennan P (2009). Quantitative analysis of DNA methylation profiles in lung cancer identifies aberrant DNA methylation of specific genes and its association with gender and cancer risk factors. Cancer Res.

[22] Pilsner JR, Hall MN, Liu X, Ilievski V, Slavkovich V, Levy D, Factor-Litvak P, Yunus M, Rahman M, Graziano JH (2012). Influence of prenatal arsenic exposure and newborn sex on global methylation of cord blood DNA. PLoS One.

[23] Kippler M, Engstrom K, Mlakar SJ, Bottai M, Ahmed S, Hossain MB, Raqib R, Vahter M, Broberg K (2013). Sex-specific effects of early life cadmium exposure on DNA methylation and implications for birth weight. Epigenetics.

[24] Sharma A, Jamil MA, Nuesgen N, Schreiner F, Priebe L, Hoffmann P, Herns S, Nothen MM, Frohlich H, Oldenburg J (2015). DNA methylation signature in peripheral blood reveals distinct characteristics of human X chromosome numerical aberrations. Clin Epigenetics.

[25] Trolle C, Nielsen MM, Skakkebaek A, Lamy P, Vang S, Hedegaard J, Nordentoft I, Orntoft TF, Pedersen JS, Gravholt CH (2016). Widespread DNA hypomethylation and differential gene expression in Turner syndrome. Sci Rep.

[26] Reizel Y, Spiro A, Sabag O, Skversky Y, Hecht M, Keshet I, Berman BP, Cedar H (2015). Gender-specific postnatal demethylation and establishment of epigenetic memory. Genes Dev.

[27] Yen A, Kellis M (2015). Systematic chromatin state comparison of epigenomes associated with diverse properties including sex and tissue type. Nat Commun.

[28] Al Tuwaijri A, Gagne-Ouellet V, Madore AM, Laprise C, Naumova AK (2016). Local genotype influences DNA methylation at two asthma-associated regions, 5q31 and 17q21, in a founder effect population. J Med Genet.

[29] Martinez F, Rosello M, Mayo S, Monfort S, Oltra S, Orellana C (2014). Duplication at Xq13.3-q21.1 with syndromic intellectual disability, a probable role for the ATRX gene. Am J Med Genet A.

[30] Linhares ND, Valadares ER, da Costa SS, Arantes RR, de Oliveira LR, Rosenberg C, Vianna-Morgante AM, Svartman M (2016). Inherited Xq13.2-q21.31 duplication in a boy with recurrent seizures and pubertal gynecomastia: clinical, chromosomal and aCGH characterization. Meta Gene.

[31] Giorgio E, Brussino A, Biamino E, Belligni EF, Bruselles A, Ciolfi A, Caputo V, Pizzi S, Calcia A, Di Gregorio E (2017). Exome sequencing in children of women with skewed X-inactivation identifies atypical cases and complex phenotypes. Eur J Paediatr Neurol.

[32] Hu H, Haas SA, Chelly J, Van Esch H, Raynaud M, de Brouwer AP, Weinert S, Froyen G, Frints SG, Laumonnier F (2016). X-exome sequencing of 405 unresolved families identifies seven novel intellectual disability genes. Mol Psychiatry.

[33] Coriell Institute for Medical Research. https://www.coriell.org/. Accessed 1 Nov 2017.

[34] Berlivet S, Moussette S, Ouimet M, Verlaan DJ, Koka V, Al Tuwaijri A, Kwan T, Sinnett D, Pastinen T, Naumova AK (2012). Interaction between genetic and epigenetic variation defines gene expression patterns at the asthma-associated locus 17q12-q21 in lymphoblastoid cell lines. Hum Genet.

[35] Barndorff-Nilesen OE, Cox DR (1994). Inference and asymptotics.

[36] Baumann C, Schmidtmann A, Muegge K, De La Fuente R (2008). Association of ATRX with pericentric heterochromatin and the Y chromosome of neonatal mouse spermatogonia. BMC Mol Biol.

[37] Sarma K, Cifuentes-Rojas C, Ergun A, Del Rosario A, Jeon Y, White F, Sadreyev R, Lee JT (2014). ATRX directs binding of PRC2 to Xist RNA and Polycomb targets. Cell.

[38] Watson LA, Goldberg H, Berube NG (2015). Emerging roles of ATRX in cancer. Epigenomics.

[39] Fukuda A, Mitani A, Miyashita T, Sado T, Umezawa A, Akutsu H (2016). Maintenance of Xist imprinting depends on chromatin condensation state and Rnf12 dosage in mice. PLoS Genet.

[40] Wang F, Shin J, Shea JM, Yu J, Boskovic A, Byron M, Zhu X, Shalek AK, Regev A, Lawrence JB, et al. Regulation of X-linked gene expression during early mouse development by Rlim. elife. 2016;5. 10.7554/eLife.19127 PMC5059138 27642011

[41] Balaton BP, Cotton AM, Brown CJ (2015). Derivation of consensus inactivation status for X-linked genes from genome-wide studies. Biol Sex Differ.

[42] Payer B, Naumova AK, Taketo T (2016). Epigenetic regulation of X-chromosome inactivation. Epigenetics in human reproduction and development.

[43] Ronen D, Benvenisty N (2014). Sex-dependent gene expression in human pluripotent stem cells. Cell Rep.

[44] Bermejo-Alvarez P, Rizos D, Rath D, Lonergan P, Gutierrez-Adan A (2010). Sex determines the expression level of one third of the actively expressed genes in bovine blastocysts. Proc Natl Acad Sci U S A.

[45] Gross N, Kropp J, Khatib H (2017). Sexual dimorphism of miRNAs secreted by bovine in vitro-produced embryos. Front Genet.

[46] Lowe R, Gemma C, Rakyan VK, Holland ML (2015). Sexually dimorphic gene expression emerges with embryonic genome activation and is dynamic throughout development. BMC Genomics.

[47] Grafodatskaya D, Chung BH, Butcher DT, Turinsky AL, Goodman SJ, Choufani S, Chen YA, Lou Y, Zhao C, Rajendram R (2013). Multilocus loss of DNA methylation in individuals with mutations in the histone H3 lysine 4 demethylase KDM5C. BMC Med Genet.

[48] Cotton AM, Ge B, Light N, Adoue V, Pastinen T, Brown CJ (2013). Analysis of expressed SNPs identifies variable extents of expression from the human inactive X chromosome. Genome Biol.

[49] Blewitt ME, Vickaryous NK, Hemley SJ, Ashe A, Bruxner TJ, Preis JI, Arkell R, Whitelaw E (2005). An N-ethyl-N-nitrosourea screen for genes involved in variegation in the mouse. Proc Natl Acad Sci U S A.

[50] Juriloff DM, Harris MJ (2012). Hypothesis: the female excess in cranial neural tube defects reflects an epigenetic drag of the inactivating X chromosome on the molecular mechanisms of neural fold elevation. Birth Defects Res A Clin Mol Teratol.

[51] Robinson WH, Cotton AM, Penaherrera MS, Peeters SB, Brown CJ, Naumova AK, Greenwood CM (2013). X chromosome inactivation. Epigenetics and complex traits.

[52] Forejt J, Gregorova S, Goetz P (1981). XY pair associates with the synaptonemal complex of autosomal male-sterile translocations in pachytene spermatocytes of the mouse (Mus musculus). Chromosoma.

[53] Homolka D, Ivanek R, Capkova J, Jansa P, Forejt J (2007). Chromosomal rearrangement interferes with meiotic X chromosome inactivation. Genome Res.

[54] Turner JM (2007). Meiotic sex chromosome inactivation. Development.

[55] Fayer S, Yu Q, Kim J, Moussette S, Camerini-Otero RD, Naumova AK (2016). Robertsonian translocations modify genomic distribution of gammaH2AFX and H3.3 in mouse germ cells. Mamm Genome.

[56] Larney C, Bailey TL, Koopman P (2014). Switching on sex: transcriptional regulation of the testis-determining gene Sry. Development.

[57] Sekido R (2014). The potential role of SRY in epigenetic gene regulation during brain sexual differentiation in mammals. Adv Genet.

[58] Li Y, Kido T, Garcia-Barcelo MM, Tam PK, Tabatabai ZL, Lau YF (2015). SRY interference of normal regulation of the RET gene suggests a potential role of the Y-chromosome gene in sexual dimorphism in Hirschsprung disease. Hum Mol Genet.

[59] Lin YN, Roy A, Yan W, Burns KH, Matzuk MM (2007). Loss of zona pellucida binding proteins in the acrosomal matrix disrupts acrosome biogenesis and sperm morphogenesis. Mol Cell Biol.

[60] Verhulst EC, van de Zande L (2015). Double nexus––Doublesex is the connecting element in sex determination. Brief Funct Genomics.

[61] Ledon-Rettig CC, Zattara EE, Moczek AP (2017). Asymmetric interactions between doublesex and tissue- and sex-specific target genes mediate sexual dimorphism in beetles. Nat Commun.

