# Supplementary material for: X chromosome dosage and presence of SRY shape sex-specific differences in DNA methylation at an autosomal region in human cells
Source: Biol Sex Differ. 2018 Feb 20;9:10. doi: 10.1186/s13293-018-0169-7 (PMC5819645; doi:10.1186/s13293-018-0169-7)
Supplement: Supplementary file 1 — Figure S1. Experimental design. (DOCX 18 kb) [file 13293_2018_169_MOESM1_ESM.docx]

**Additional file 1. Figure S1. Experimental design**
