# Supplementary material for: X chromosome dosage and presence of SRY shape sex-specific differences in DNA methylation at an autosomal region in human cells
Source: Biol Sex Differ. 2018 Feb 20;9:10. doi: 10.1186/s13293-018-0169-7 (PMC5819645; doi:10.1186/s13293-018-0169-7)
Supplement: Supplementary file 2 — Table S1. Primers used in the study. (DOCX 12 kb) [file 13293_2018_169_MOESM2_ESM.docx]

**Additional file 2. Table S1**. Primers used in the study

| **Assay** | **Region** | **Forward primer 5’-3’** | **Reverse primer 5’-3’** | **Sequencing primer** | **Coordinates (hg19))** |
| --- | --- | --- | --- | --- | --- |
| Bisulfite pyrosequencing | *ZPBP2* DMR | Biotin-GTGACGTACTAGCAACGAGAGAGTAAGGGGTTAGGAG | TAGCAGGATACGACTATCTCCCCACCTCCTCCACAATA | ATCCCCTCCCCTACCC (reverse) | Chr17: 38024296-38024536 |
| Genotyping | *SRY* | TCTTGAGTGTGTGGCTTTCG | TACAGGCCATGCACAGAGAG | - | ChrY: 2787078-2787257 |
