# Supplementary material for: X chromosome dosage and presence of SRY shape sex-specific differences in DNA methylation at an autosomal region in human cells
Source: Biol Sex Differ. 2018 Feb 20;9:10. doi: 10.1186/s13293-018-0169-7 (PMC5819645; doi:10.1186/s13293-018-0169-7)
Supplement: Supplementary file 3 — Table S2. Fibroblast cell lines used for exploratory analysis. (DOCX 16 kb) [file 13293_2018_169_MOESM3_ESM.docx]

**Additional file 3. Table S2.** Fibroblast cell lines used for exploratory analysis

| **Type** | **Phenotype** | **Coriell ID** | **Age**  **(years)** | **Sex** | ***SRY**** | **Passage** | **Karyotype** |
| --- | --- | --- | --- | --- | --- | --- | --- |
| 46,XY | Apparently healthy | GM07753 | 17 | M | **+** | 6 | 46,XY |
| 46,XY | Apparently healthy | GM02936 | 0  (20 days) | M | **+** | 6 | 46,XY |
| 46,XY | Apparently healthy | GM03348 | 10 | M | **+** | 7 | 46,XY |
| 46,XY | Apparently healthy | GM17333 | 41 | M | **+** | 9 | 46,XY |
| 46,XY | Apparently healthy | GM04501 | 19 | M | **+** | 12 | 46,XY.arr 2q23.1(148922880-149041426)x3 |
| 46,XY | Apparently healthy | GM07492 | 17 | M | **+** | 3 | 46,XY |
| 46,XY | Apparently healthy | GM04502 | 19 | M | **+** | No data | 46,XY |
| 46,XX | Apparently healthy | GM00037 | 18 | F | **−** | 16 | 46,XX |
| 46,XX | Apparently healthy | GM01652 | 11 | F | **−** | 11 | 46,XX |
| 46,XX | Apparently healthy | GM00038 | 26 | F | **−** | 10 | 46,XX |
| 46,XX | Apparently healthy | GM17332 | 35 | F | **−** | 9 | 46,XX |
| 46,XX | Apparently healthy | GM07525 | 22 | F | **−** | 6 | 46,XX |
| 46,XX | Apparently healthy | GM07545 | 22 | F | **−** | 5 | 46,XX |
| 46,XX | Apparently healthy | GM00023 | 31 | F | **−** | 8 | 46,XX |
| 46,XX | Apparently healthy | GM17375 | 19 | F | **−** | 6 | 46,XX |
| 46,XX | Apparently healthy | GM17071 | 18 | F | **−** | 11 | 46,XX |
| 46,XX | Apparently healthy | GM07532 | 16 | F | **−** | 7 | 46,XX |
| 46,XX | Apparently healthy | GM07522 | 19 | F | **−** | 2 | 46,XX |
| 46,XX | Apparently healthy | GM04505 | 20 | F | **−** | 10 | 46,XX |
| 46,XX | Apparently healthy | GM04390** | 23 | F | **−** | 9 | 46,XX |
| 46,XX | XX male syndrome | GM02626 | 23 | M | **+** | 4 | 46,XX.ish der(X)t(X;Y)(p22.3;p11.3)(SRY+,DXZ1+).arr Yp11.31p11.2(2710425-5680476)x1 |
| 46,XX | XX male syndrome | GM02670 | 19 | M | **+** | 7 | 46,XX.ish der(X)t(X;Y)(p22.3;p11.3)(SRY+,DXZ1+).arr Yp11.31p11.2(2710425-5680476)x1 |
| 46,XX | XX male syndrome | GM01889 | 24 | M | **−** | 3 | 46,XX |
| 49,XXXXY | XXXXY male syndrome | GM00157 | 28 | M | **+** | 12 | 49,XXXXY,t(4;11)(q35;q23) |
| 49,XYYYY | XYYYY syndrome | GM11420 | 23 | M | **+** | 2 | 49,XYYYY |
| 46,XY | 46,XY sex reversal 1;SRXY1 | GM00048 | 1 | F | **+** | 7 | 46,XY (ZFY+) |
| 46,XY | 46,XY sex reversal 1;SRXY1 | GM01628 | 1 | F | **+** | 2 | 46,XY (H-Y antigen positive) |
| 46,XY | 46,XY sex reversal 1;SRXY1 | GM03368 | 17 | F | **+** | 4 | 46,XY |
| 46,XY | Androgen insensitivity | GM02717 | 14 | F | **+** | 3 | 46,XY |
| 45,X | Turner syndrome | GM01176 | 8 | F | **−** | 5 | 45,X |
| 45,X | Turner syndrome | GM00857 | 0 (1 day) | F | **−** | 5 | 45,X.arr Xp22.33q28(108464-154887040)x1 |
| 45,X | Turner syndrome | GM00562 | 14 | F | **−** | 4 | 45,X[46]/46,XX[4] |
| 45,X | Turner syndrome | GM02668 | 41 | Amb. | **+** | 4 | 46,X,del(Y)(pter>q11.2:)[77]/45,X[33] |
| 45,X | Turner syndrome | GM03774 | 16 | F | + | 2 | 46,fra(X)(q27.3),Y[36]/46,X,i(Y)(q10)[14].arr Yp11.31(2758970-3076489)x1˜2,Yp11.2(7023887-8627175)x1˜2 |
| 45,X | Turner syndrome | GM01941 | 13 | F | + | 3 | 45,X[5]/46,X,idic(Y)(q12)[5].ish Xp11.1q11.1(DXZ1x1),Xq28(Z43206x1),idic(Y)(q12)(SRY+,DYZ3+,DYZ1 enh,Z43206-,DYZ3+,SRY+).arr Yp11.31(2840527-2962962)x0 |
| 45,X | Aneupolid chromosome number, non-trisomic | GM01723 | 23 | F | **_** | 6 | 45,X |
| 46,XY | Androgen insensitivity | GM02717 | 14 | F | **+** | 2 | 46,XY |

* *SRY* genotype information was taken from the Coriell website; if no information was available, samples were genotyped by PCR (primers listed in Table S1) (Information from <https://catalog.coriell.org>)
