# Supplementary material for: X chromosome dosage and presence of SRY shape sex-specific differences in DNA methylation at an autosomal region in human cells
Source: Biol Sex Differ. 2018 Feb 20;9:10. doi: 10.1186/s13293-018-0169-7 (PMC5819645; doi:10.1186/s13293-018-0169-7)
Supplement: Supplementary file 4 — Table S3. Fibroblast cell lines used for mapping of the X-linked modifier of methylation. (DOCX 14 kb) [file 13293_2018_169_MOESM4_ESM.docx]

**Additional file 4. Table S3**. Fibroblast cell lines used for mapping of the X-linked modifier of methylation

| **Type** | **Phenotype** | **Coriell ID** | **Age**  **(years)** | **Sex** | ***SRY* genotype** | **Karyotype** |
| --- | --- | --- | --- | --- | --- | --- |
| 45,X | Turner syndrome | GM00857 | 0  (1 day) | F | **−** | 45,X.arr Xp22.33q28(108464-154887040)x1 |
| 45,X | Turner syndrome | GM00562 | 14 | F | **−** | 45,X[46]/46,XX[4] |
| 45,X | Turner syndrome | GM01176 | 8 | F | **−** | 45,X |
| 45,X | Aneupolid chromosome number, non-trisomic | GM01723 | 23 | F | **_** | 45,X |
| 46,X,i(Xq) | Turner syndrome | GM02595 | 33 | F | **−** | 46,X,i(X)(qter>q10::q10>qter) |
| 45,X/  46,X,idic(X) | Turner syndrome | GM00339 | 25 | F | **−** | 45,X,t(2;4)(2qter>2p13::4q31>4qter;4pter>4q31::2p13>2pter)[28]/46,X,idic(X)(qter>p11::p11>qter)[15]/45,X[5}/47,X,idic(X)(p11),+idic(X)(p11)[2] |
| 46,X,i(Xq) | Turner syndrome | GM00735 | 17 | F | **−** | 45,X.arr Xp22.33p11.1(108464-52927933)x1, Xp11.22q28(53086710-154887040)x2~3, 8p23.1(7254762-7825360)x1,22q12.3q13.33(32495977-49406499)x2~3 |
| 46,X,idic(X) | Turner syndrome | GM00088 | 19 | F | **-** | 46,X,idic(X)(p11.2).arr Xp22.33p11.21(108464-56474519)x1,Xp11.21q28(56,486,208-154,887,040)x3,5p15.2p15.1(14450134-15493849)x3,12p11.1(33420095-34694301)x3 |
| 47,XXX | Abortus; clinically normal phenotype | GM04626 | 21 fw | F | **−** | 47,XXX.arr(X)x3,(Y)x0,15q11.2(18522237-21038975)x1 |
| 47,XXX |  | GM00254 | 10 fw | F | **−** | 47,XXX[48]/46,XX[2] |
| 48,XXX | Trisomy 18 | GM03623 | nd | F | **−** | 48,XXX,+18.arr(X)x3,(Y)x0,1q31.3(195005233-195168376)x1,(18)x3 |
| DEL |  | GM03624 | 23 | F | **−** | 46,X,del(X)(q25).arr Xq25q28(129376097-154887040)x1 |
| DEL |  | GM03923 | 25 | F | **−** | 46,X,del(X)(pter>q13::q22>qter) |
| DEL |  | GM07148 | 18 | F | **−** | 46,X,del(X)(q22.3).arr Xq22.3q28(108841425-154887040)x1 |
| DEL |  | GM07213 | 14 | F | **−** | 46,X,idic(X)(q28)[15]/47,X,idic(X)(q28),+7[3]/45,X[2].arr Xp22.33q27.1(273978-138007119)x2~3,Xq27.1q27.2(138128434-141477946)x4~5,Xq27.2q28(141481461-154887040)x1,15q13.3(30245952-30644762)x1,17q12(31461228-31653797)x4 |

Fw- fetal weeks
