# Supplementary material for: X chromosome dosage and presence of SRY shape sex-specific differences in DNA methylation at an autosomal region in human cells
Source: Biol Sex Differ. 2018 Feb 20;9:10. doi: 10.1186/s13293-018-0169-7 (PMC5819645; doi:10.1186/s13293-018-0169-7)
Supplement: Supplementary file 5 — Figure S2. Sodium bisulfite sequencing methylation analysis of 51 CGs across the ZPBP2 promoter region shows higher methylation levels in fibroblast cell lines with two and three copies of the Xq arm. A: location of the 51 CGs interrogated using the sodium bisulfite sequencing assay shown in the context of the UCSC browser (hg19). B: heatmap representing mean methylation levels for each of the 51 CGs in cell lines with one X chromosome (two fibroblast cell lines with karyotype 45,X and no SRY region) and two or three copies of Xq (data from four fibroblast cell lines with karyotype 46,XX and two fibroblast cell lines from Turner syndrome patients with karyotype 46,i(Xq)). The black box beneath the heatmap shows the location of the 10 CGs analyzed by the pyrosequencing assay. The color scale for percent methylation is shown on the right. (DOCX 292 kb) [file 13293_2018_169_MOESM5_ESM.docx]

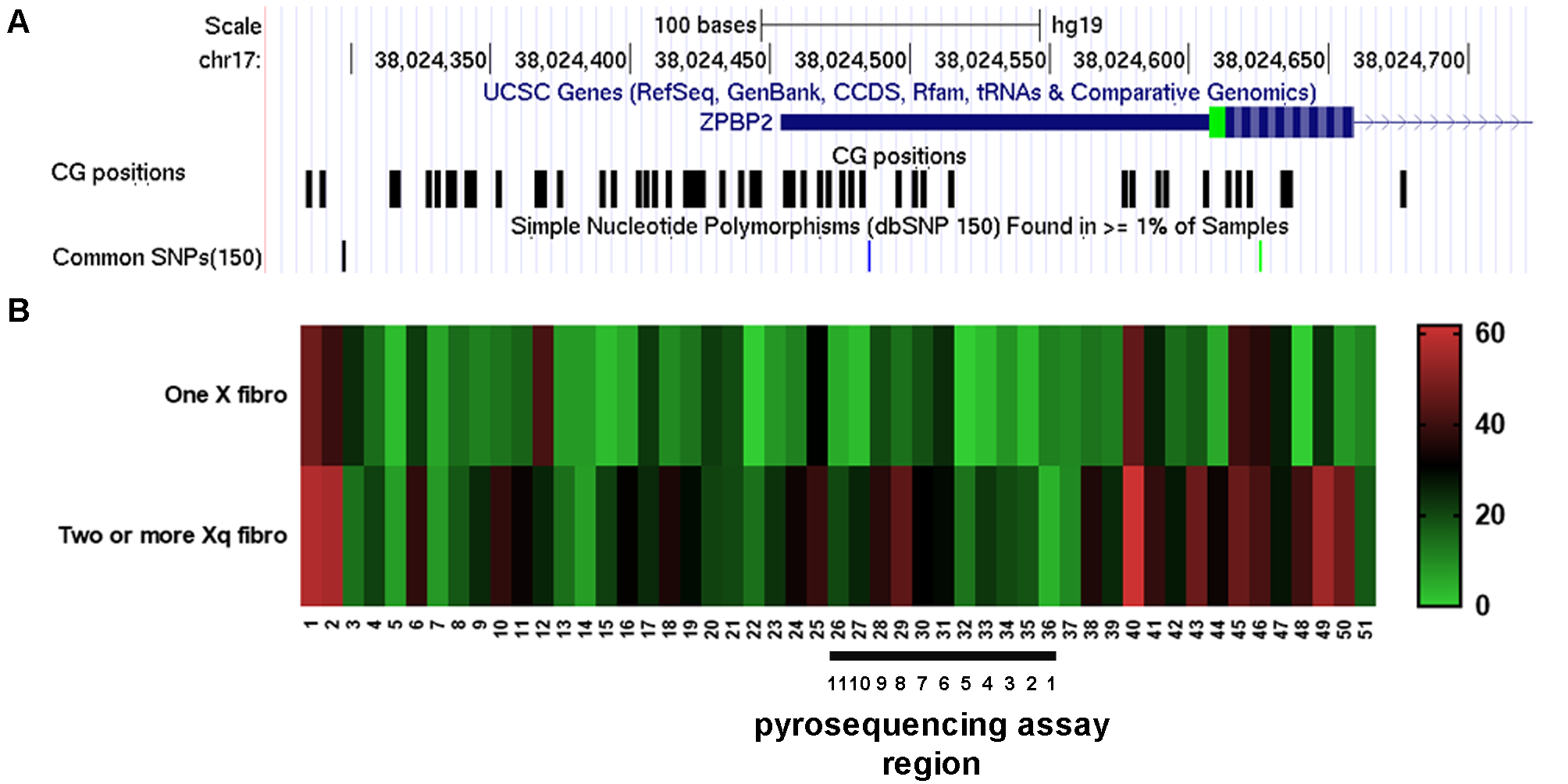


**Additional file 5. Fig.S2. Sodium bisulfite sequencing methylation analysis of 51 CGs across the *ZPBP2* promoter region shows higher methylation levels in fibroblast cell lines with 2 and 3 copies of the Xq arm.**

**A:** location of the 51 CGs interrogated using the sodium bisulfite sequencing assay shown in the context of the UCSC browser (hg19). **B**: heatmap representing mean methylation levels for each of the 51 CGs in cell lines with one X chromosome (2 fibroblast cell lines with karyotype 45,X and no *SRY* region) and two or three copies of Xq (data from 4 fibroblast cell lines with karyotype 46,XX and 2 fibroblast cell lines from Turner syndrome patients with karyotype 46,i(Xq)). The black box beneath the heatmap shows the location of the 10 CGs analysed by the pyrosequencing assay. The color scale for % methylation is shown on the right.
