# Supplementary material for: X chromosome dosage and presence of SRY shape sex-specific differences in DNA methylation at an autosomal region in human cells
Source: Biol Sex Differ. 2018 Feb 20;9:10. doi: 10.1186/s13293-018-0169-7 (PMC5819645; doi:10.1186/s13293-018-0169-7)
Supplement: Supplementary file 6 — Table S4. Methylation percentages at the analyzed ZPBP2 DMR CGs from human non-transformed fibroblast cell lines, and the first PC of these values. (DOCX 14 kb) [file 13293_2018_169_MOESM6_ESM.docx]

**Additional file 6: Table S4. Methylation percentages at the analyzed *ZPBP2* DMR CGs from human non-transformed fibroblast cell lines, and the first PC of these values.**

| **Fibroblast cell line ID** | **ZPBP2 DMR methylation (pyrosequencing assay)** | | | | | | | | | | **First PC** |
| --- | --- | --- | --- | --- | --- | --- | --- | --- | --- | --- | --- |
|  | **CG2** | **CG3** | **CG4** | **CG5** | **CG6** | **CG7** | **CG8** | **CG9** | **CG10** | **CG11** |  |
| GM04505 | 26 | 74 | 24 | 16 | 34 | 19 | 23 | 21 | 13 | 23 | 24.69 |
| GM07522 | 22 | 25 | 19 | 11 | 27 | 23 | 25 | 26 | 16 | 20 | 1.935 |
| GM07532 | 31 | 25 | 32 | 17 | 36 | 24 | 31 | 25 | 20 | 27 | 17.71 |
| GM00023 | 24 | 15 | 22 | 15 | 22 | 14 | 16 | 20 | 16 | 20 | -9.926 |
| GM07525 | 21 | 9 | 36 | 8 | 17 | 10 | 19 | 20 | 9 | 16 | -15.03 |
| GM07545 | 19 | 12 | 16 | 49 | 22 | 13 | 26 | 25 | 11 | 12 | -3.716 |
| GM17071 | 44 | 25 | 52 | 36 | 51 | 33 | 45 | 58 | 28 | 20 | 58.07 |
| GM17332 | 45 | 11 | 33 | 10 | 42 | 28 | 43 | 32 | 19 | 39 | 26.73 |
| GM17375 | 22 | 9 | 28 | 18 | 32 | 14 | 20 | 28 | 22 | 35 | -0.1325 |
| GM00038 | 29 | 49 | 27 | 50 | 34 | 26 | 54 | 21 | 13 | 17 | 37.74 |
| GM01652 | 22 | 13 | 16 | 11 | 17 | 14 | 24 | 16 | 11 | 18 | -15.71 |
| GM07492 | 33 | 11 | 14 | 8 | 47 | 10 | 17 | 30 | 13 | 15 | -1.665 |
| GM17333 | 18 | 19 | 14 | 9 | 18 | 22 | 26 | 15 | 10 | 24 | -11.68 |
| GM03348 | 67 | 56 | 22 | 14 | 32 | 21 | 21 | 18 | 15 | 22 | 29.31 |
| GM07753 | 21 | 9 | 11 | 8 | 24 | 15 | 21 | 20 | 13 | 25 | -15.59 |
| GM03774 | 10 | 9 | 11 | 16 | 15 | 8 | 11 | 13 | 6 | 9 | -32.33 |
| GM01941 | 37 | 21 | 26 | 22 | 33 | 18 | 31 | 17 | 16 | 15 | 9.870 |
| GM02668 | 13 | 10 | 12 | 6 | 37 | 14 | 28 | 16 | 9 | 15 | -13.73 |
| GM01176 | 29 | 15 | 13 | 7 | 16 | 18 | 17 | 19 | 14 | 18 | -14.23 |
| GM01723 | 16 | 15 | 14 | 7 | 18 | 9 | 13 | 14 | 8 | 24 | -24.66 |
| GM00857 | 30 | 16 | 15 | 6 | 17 | 10 | 13 | 16 | 8 | 16 | -19.42 |
| GM01628 | 31 | 18 | 13 | 5 | 30 | 12 | 25 | 20 | 16 | 18 | -5.764 |
| GM02717 | 31 | 15 | 11 | 4 | 28 | 8 | 20 | 27 | 16 | 19 | -9.705 |
| GM00048 | 24 | 11 | 10 | 6 | 15 | 9 | 14 | 11 | 7 | 10 | -28.10 |
| GM02670 | 13 | 12 | 14 | 11 | 17 | 9 | 14 | 23 | 12 | 13 | -23.46 |
| GM01889 | 55 | 52 | 42 | 26 | 69 | 64 | 53 | 53 | 41 | 22 | 90.33 |
| GM02626 | 34 | 24 | 20 | 9 | 24 | 11 | 17 | 17 | 13 | 14 | -6.765 |
| GM00157 | 29 | 16 | 20 | 17 | 19 | 11 | 15 | 24 | 13 | 18 | -10.16 |
| GM011420 | 9 | 6 | 10 | 7 | 18 | 7 | 9 | 13 | 11 | 15 | -34.63 |
