# Supplementary material for: X chromosome dosage and presence of SRY shape sex-specific differences in DNA methylation at an autosomal region in human cells
Source: Biol Sex Differ. 2018 Feb 20;9:10. doi: 10.1186/s13293-018-0169-7 (PMC5819645; doi:10.1186/s13293-018-0169-7)
Supplement: Supplementary file 7 — Figure S3. Scatter plot of the first (PC1) and second (PC2) eigenvectors from a principal component analysis of methylation levels at 10 CpGs near the ZPBP2 transcriptional start site. The first principal component explains 59.2% of the variance in methylation levels. Points are colored by groups defined by presence of SRY and the number of X chromosomes. (DOCX 118 kb) [file 13293_2018_169_MOESM7_ESM.docx]

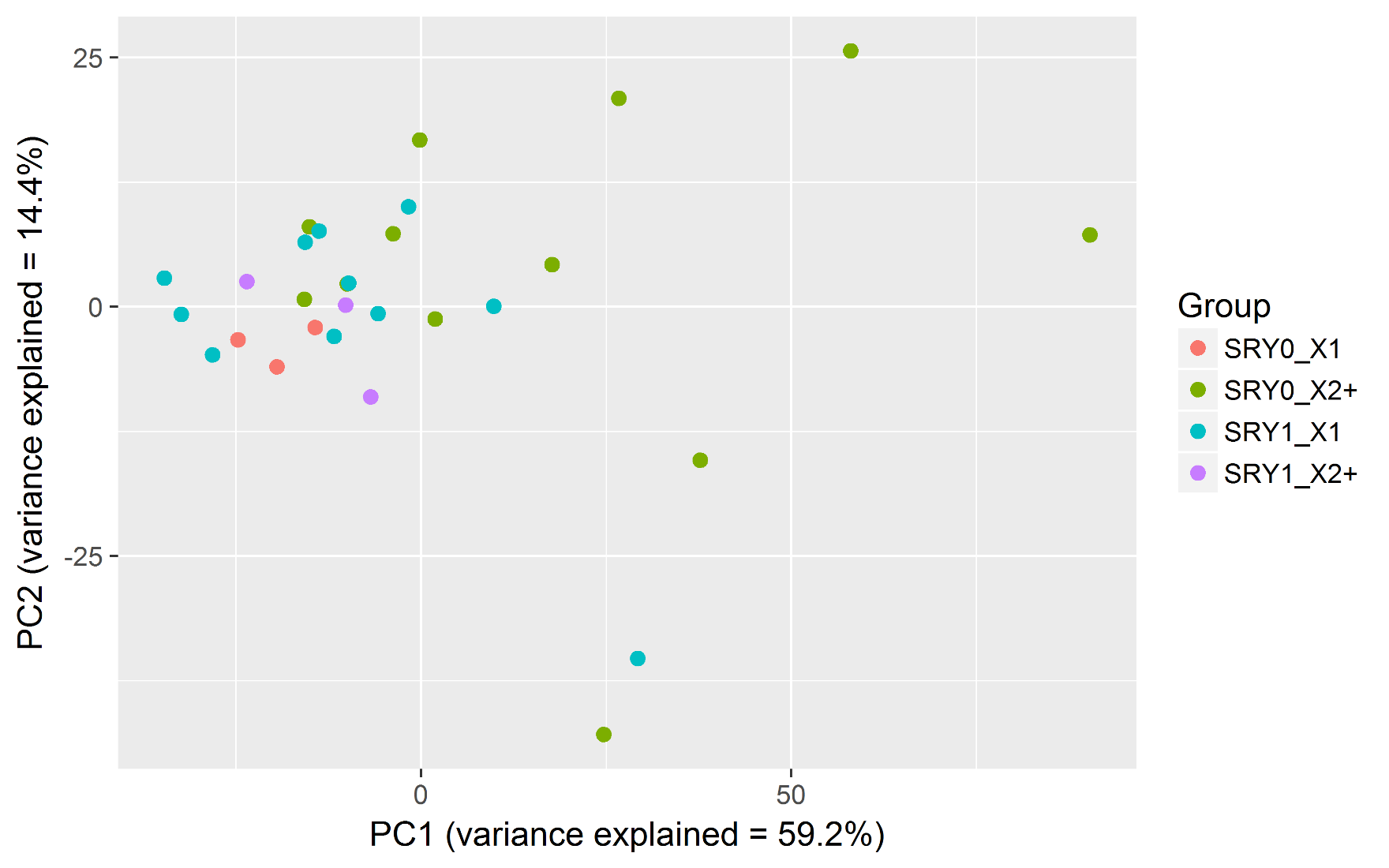


**Additional file 7: Figure S3.** Scatter plot of the first (PC1) and second (PC2) eigenvectors from a principal component analysis of methylation levels at 10 CpGs near the *ZPBP2* transcriptional start site. The first principal component explains 59.2% of the variance in methylation levels. Points are coloured by groups defined by presence of SRY and the number of X chromosomes.
