# Supplementary material for: X chromosome dosage and presence of SRY shape sex-specific differences in DNA methylation at an autosomal region in human cells
Source: Biol Sex Differ. 2018 Feb 20;9:10. doi: 10.1186/s13293-018-0169-7 (PMC5819645; doi:10.1186/s13293-018-0169-7)
Supplement: Supplementary file 8 — Table S5. Univariate analysis results, full model multivariate analysis results, as well as post hoc power calculations. (DOCX 14 kb) [file 13293_2018_169_MOESM8_ESM.docx]

**Additional file 8: Table S5. Univariate analysis results, full model multivariate analysis results, as well as *post hoc* power calculations.**

| **Covariate** | **Univariate Model** | | | **Full Model** | | | **Post hoc Power**  **(Full Model)^2^** |
| --- | --- | --- | --- | --- | --- | --- | --- |
|  | **Estimate** | **Std. Error** | **p-value** | **Estimate** | **Std. Error** | **p-value** |  |
| Sex: Male | 1.85 | 11.65 | 0.88 | 37.53 | 15.66 | 0.026 | 0.669 |
| Age | 0.24 | 0.52 | 0.65 | -0.73 | 0.61 | 0.25 | 0.221 |
| Passage Number | 2.01 | 1.71 | 0.25 | 1.12 | 1.89 | 0.56 | 0.091 |
| One or More Y chromosomes | -14.18 | 11.06 | 0.21 | -15.93 | 18.82 | 0.41 | 0.135 |
| Presence of SRY | -21.32 | 9.72 | 0.037 | 8.14 | 21.80 | 0.71 | N/A |
| Two or More X chromosomes | 23.80 | 9.50 | 0.019 | 39.62 | 17.00 | 0.030 | N/A |
| Interaction: SRY: two or more X^1^ | N/A | N/A | N/A | -67.15 | 26.64 | 0.020 | N/A |

The results in the univariate model are obtained by fitting linear regressions of the first PC against each variable at a time. The multivariate result was obtained from a model containing all listed variables simultaneously in the model. Post hoc power was calculated from the full model estimates using a significance threshold of 0.05. Univariate analysis with the variable “Sex” was performed with 28 samples, since sex could not be assigned for one sample. All other univariate models had a sample size of 29. The full model with all variables was performed on a sample size of 28.

^1^ Fitting an interaction without the main effects would be inappropriate and hence no results are reported the univariate columns

^2^ Post hoc power is reported in response to a reviewer request. It is left as NA for the variables involved in the significant interaction.
